# Association of Inherited Thrombophilia with Recurrent Pregnancy Loss in Palestinian Women

**DOI:** 10.1155/2011/689684

**Published:** 2011-06-14

**Authors:** N. S. Abu-Asab, S. K. Ayesh, R. O. Ateeq, S. M. Nassar, W. A. EL-Sharif

**Affiliations:** ^1^Joint Medical Program of University of New England & The University of Newcastle; and Armidale Rural Referral Hospital, Armidale, NSW 2351, Australia; ^2^Molecular Genetics Laboratory, AL-Makassed Islamic Charitable Society Hospital, Mount of Olives, Jerusalem 91194, Palestine

## Abstract

*Objective*. This study aimed at analyzing the association between recurrent pregnancy loss (RPL) and factor V G1691A (FVL), prothrombin G20210 (FII); and MTHFR C677T (MTHFR) in Palestinian women. *Method*. We studied 329 Palestinian women with RPL and/or stillbirth (SB); and compared them to 402 healthy reproductive Palestinian women. Cases and controls were tested for the above mutations. Odds ratio (OR) at confidence interval (CI) of 95% was used as a measure of association between the mutations and RPL. *Results*. Our statistical analysis showed a slightly increased association, which was not significant between FVL and RPL (OR 1.32, 95% CI 0.90–1.94), and no association between FII (OR 0.84, 95% CI 0.38–1.92), MTHFR (OR 0.58, 95% CI 0.32–1.03), and RPL. Further analysis of RPL subgroups revealed an association between FVL and first-trimester loss (OR 1.33, 95% CI 0.892–1.989), and second-trimester loss (OR 1.13, 95% CI 0.480–2.426), both were not statistically significant. Furthermore, the only statistically significant association was between FVL and SB (OR 2.0, 95% CI 1.05–3.70). *Conclusion*. Our analysis had failed to find a significant association between FVL, FII, MTHFR; and RPL in either the first or second trimester. FVL was significantly associated with fetal loss if the loss was a stillbirth.

## 1. Introduction

Molecular studies of coagulation disorders have led to the discovery of an increasing number of mutations in the genes of the factors of coagulation, termed inherited thrombophilia (IT). The most important of this group are factor V gene Leiden mutation G1691A (FVL), prothrombin G20210 (FII), homozygosity for the thermolabile of methyltetrahydrofolate reductase deficiency C677T (MTHFR), antithrombin deficiency, protein C deficiency, and protein S deficiency [1–7]. These conditions affect coagulation cascade at different stages; increasing the potential for thromboembolic diseases (TE) [4, 7].

The importance of IT in obstetrics stems from being partly responsible for up to half of maternal venous TE [2, 5], which is one of the direct leading causes of maternal mortality [2, 5, 8]. While the relationship of IT to TE is not disputed and has been confirmed by a number of studies [4, 7], the evidence is conflicting regarding the association with pregnancy complications [1, 5, 6, 8–11]. A delicate balance between coagulant and anticoagulant factors is needed to achieve a successful pregnancy [12]. A balanced system maintains the blood flow to the feto-maternal exchange unit and promotes trophoblastic proliferation [2, 5, 6]. The hypercoagulable state that occurs during pregnancy makes it tempting to postulate that pregnancy association with a thrombophilic condition may be detrimental through either repeated pregnancy loss (RPL), intrauterine fetal death (IUFD), and/or other complications such as placental abruption, intrauterine growth retardation (IUGR), and early onset of preeclampsia [2–4, 7, 8].

Researchers have been working on inherited thrombophilia to explain RPL, especially that 30–50% of RPL cases remain enigmatic [1, 13]. RPL is classically defined as three or more spontaneous fetal losses before the 20th week of pregnancy [8, 11]; however, two or more losses were considered in some studies [8]. It is a serious condition affecting 5% of pregnant women with devastating social and medical consequences [1, 2, 8].

This study aimed at testing the association between FVL, FII, MTHFR; and RPL in Palestinian women. The frequencies of these mutations were statistically compared among cases with RPL and a control group of healthy women to test whether significant differences in mutations frequencies exist between the two groups.

## 2. Materials and Methods

This study protocol was approved by UNFPA and the Research and Ethics Committee of Al-Makassed Islamic Charitable Society Hospital, Jerusalem; Palestine, and all subjects involved gave informed verbal consent at the beginning of the study.

### 2.1. Study Subjects

#### 2.1.1. Cases

 We studied 329 Palestinian females who were referred to the Molecular Genetics Laboratory at Al-Makassed Islamic Charitable Society Hospital, Jerusalem, Palestine; which is the main tertiary hospital serving the Palestinian territories. These cases were referred between January 2002 and April 2006, because of RPL and/or SB. They were referred by obstetricians from all over the Palestinian territories as part of their continuing management of the presenting condition and few were already pregnant and some were receiving empirical anticoagulant therapy. 

RPL was defined as two or more consecutive fetal losses in the first or second trimester (up to 24 weeks of pregnancy), while stillbirth (SB) as any “child” expelled from its mother at 24th week of pregnancy or more without showing any sign of life. 

The cases were Palestinian women residing in the territories who had no prior infertility, TE; or immunological diseases and had their past pregnancies confirmed by ultrasound scan. They had normal uterine cavities confirmed mainly by hysterosalpingography and had negative anticardiolipin profile. Cases with no prior normal parental karyotyping were excluded. Some cases were still being investigated for other risk factors and etiologies, for example, infections and endocrinological causes.

During the study period; there were 329 cases of RPL who fulfilled the study criteria; 267 had recurrent first-trimester abortion, 50 had recurrent second-trimester abortions, and 21 had both first and second-trimester abortions, as well as 65 who had SB, out of which 45 were associated with RPL.

#### 2.1.2. Control Group

402 healthy Palestinian women were recruited. They were recruited from women attending gynaecology clinics at various hospitals (public & NGO) in all the districts of the Palestinian territories. They were attending clinics mainly for postpartum checks and contraception but not infertility. They were 20–45 years of age, nonpregnant, and had given birth to one or more healthy infants. None had a history of fetal loss, preeclampsia, IUGR, abruption placenta, any kind of thrombosis, or known immunological disease. 

All women were living in the Palestinian territories, and detailed information about the obstetric, medical; and family history was collected by trained laboratory technicians who were aware of the purpose of the study; they used a standardized medical history form for both cases and controls. 

Cases and controls were tested for the following mutations: factor V G1691A, Prothrombin G20210; and MTHFR C677T.

### 2.2. Molecular Diagnosis

DNA was extracted from the blood specimens using the MasterPure Genomic DNA Purification Kit (Epicentre Technologies, USA) according to the standard procedure [14]. We used the amplification refractory mutation system (ARMS) as the diagnosing method. ARMS is an amplification strategy in which a polymerase chain reaction (PCR) primer is designed in such a way in order to discriminate among templates that differ by a single nucleotide residue [15, 16].

Genomic DNA (0.2 *μ*g) and common normal and mutated primers (0.1 *μ*g) were used in the reaction containing 0.625 U of peQlab Polymerase enzyme and MgCl_2_ at a concentration of 1.5 mM in a final volume of 25 uL. PCR conditions for factor V G1691A were as follows: initial denaturation at 98°C for 2 min, 98°C for 20 sec, 56°C for 35 sec, 72°C for 35 sec, for 28 cycles, and final extension at 72°C for 5 min.

PCR conditions for prothrombin G20210A were as follows: initial denaturation at 94°C for 5 min, 94°C for 15 sec, 56°C for 15 sec, 72°C for 30 sec, for 33 cycles, and final extension at 72°C for 10 min.

PCR conditions for MTHFR C677T were as follows: initial denaturation at 94°C for 5 min, 94°C for 30 sec, 57°C for 30 sec, 72°C for 30 sec, for 27 cycles, and final extension at 72°C for 5 min.

In order to increase specificity of bands, formamide was added at a concentration of 3% to the MTHFR C677T reaction mixture and at 2% to the prothrombin G20210A mixture. Each amplified PCR product was analyzed using 2% agarose gel electrophoresis (Tech Comp, Ltd., USA), and stained with SYBR Green stain. Bands were visualized under UV illuminator, and photographed with a Polaroid camera (Fuji Photo Film Co., Japan).

### 2.3. Statistical Analysis

Exact logistic regression was performed using Stata version 10 software. The Odds Ratio at 95% confidence interval was used as a measure of association between genotypes and RPL. Statistical significance was set at *P* < .05.

## 3. Results

Both cases and controls were Palestinian women living in all districts of the Palestinian territories. The mean age of the cases and the controls was not significantly different but their parity differed significantly, the cases had a parity of 1.16 and the controls had a parity of 3.15 (*P* = .000) reflecting the effect of RPL on achieving the desired fertility. Consanguinity was significantly greater in the cases compared to the controls; 33.1% versus 24.6% (*P* = .006). Other risk factors and variables were compared, and the results are summarized in Table 1.

There were 77 FVL mutations in the case group representing a prevalence of 23.4%, and there were 73 (18.2%) carriers in the controls. 70 out of 309 (22.7%) cases with RPL were carriers of FVL mutations resulting in OR 1.32 (95% CI: 0.89–1.94). 11 out of 309 (3.6%) cases with RPL were carriers of FII mutations compared to 17 out of the 402 (4.2%) controls, OR 0.84 ( 95% CI: 0.35–1.92). 20 out of the 309 (6.5%) cases with RPL were homozygous for MTHFR compared to 43 out of 402 (10.4%) of the control group, OR 0.58 (95% CI = 0.31–1.03). The results are summarized in Table 2. Cases and controls were classified according to their consanguinity status, numbers, and prevalence for each category are displayed in Table 2.

Further association was tested after dividing the cases into 3 subgroups: groupA included 267 women with repeated first-trimester miscarriage, B included 50 women with repeated second-trimester miscarriage, and C included 65 women with SB (Table 3).

FVL was higher in the three subgroups of RPL/SB, with OR in the first-trimester subgroup 1.33 (95% CI: 0.892–1.989), the second-trimester subgroup OR 1.13 (95% CI: 0.480–2.426), and the SB subgroup OR 2.0 (*P* = .028, 95% CI: 1.053–3.701). Results are summarized in Table 3. The results for FII and MTHFR and the different subgroups are also summarized in Table 3.

Furthermore, it was of importance to study the association of the three mutations to cases with only first-trimester miscarriages and without a prior successful pregnancy (primary RPL), given that this group seem to display different degrees of association to clinical risk factors in case-control studies and behave differently in treatment trials. Our results did not yield significant association between the three mutations and primary RPL. The results are summarized in Table 3.

The prevalence of the genotypes and the alleles of the three mutations was analyzed separately; the results are summarized in Table 4.

## 4. Discussion

The study subjects were of similar ethnic and social background and were living in the different districts of the Palestinian territories. We compared some characteristics, for example, age, parity, consanguinity; and family history of TE, which are considered by many researchers as the most important risk factors. It was thought that alcohol or drug use were irrelevant in the context of Palestinian women in the reproductive age. Smoking role in RPL is not yet clearly defined, and due to extremely low rate of smoking among young Palestinian women [17]; we did not include it as a confounder in our study. 

 Some researchers suggested that RPL is a multifactorial/polygenetic condition [11], and several of these risk factors are insufficient independently for leading to RPL. It is when several intrinsic and extrinsic factors come together in the same individual that the risk exceeds the threshold and disease develops [11]. Hence, our decision is to study and report cases who were being investigated for other risk factors and etiologies, for example, infections and endocrinological causes. 

Our analysis indicates a high prevalence of FVL (18.2%) in the normal controls with an allele frequency (AF) of 9.6% (Table 4). These figures fall within the range of related ethnic groups such as Israeli Arabs (25.7%, AF 13.6%) [18], Jordanians (12.3–15%) [19, 20], and Lebanese (12–14.4%) [21–23]. They are also congruent with other studies showing that eastern Mediterranean populations have a high prevalence of FVL [23], for example, Greek Cypriots (13.3%) [24] and Syrians (13.6%) [23]. The narrow difference in frequencies among these populations suggests a single origin of the mutation, which has been proven by haplotype analysis [25].

The high prevalence of FVL seen in our study has mitigated the significance of association with RPL. It is not surprising that the high prevalence of FVL was correlated with a consanguinity rate of 25% in the normal controls and 33% in the RPL group, which could have enhanced inheritance of the mutated alleles in this population. Studying association of consanguineous cases and controls revealed O.R. 1.16 (95% CI 0.32–3.73), while studying association of non-consanguineous cases and controls revealed O.R. 0.79 (95% CI 0.29–1.91). 

In this background of high consanguinity rates, we had to analyze and compare the prevalence of the genotypes and the alleles of the three mutations (illustrated in Table 4). The detailed analysis of the different alleles of the three mutations was in agreement with Hardy-Weinberg equilibrium. 

Consanguinity among Palestinians ranges from 24 to 45% [26], and it appears to be the strongest risk factor for reproductive wastage after age and parity in Palestinian women (Assaf S., pers. com.). In Egypt, first-cousin couples were found to have higher likelihood of repeated miscarriages, stillbirths, and neonatal deaths than couples who were less related [27]. 

Few case-control studies and meta-analysis had shown a high prevalence of FVL in women with RPL [3, 10, 28–31]. A systematic review of the literature found an OR for the association between RPL and FVL of 2.0 (95% CI 1.5–2.7; *P* < .001) [3]. Reznikoff-Etiévant et al. evaluated 260 consecutive white women who had experienced two or more unexplained losses at less than 10 weeks gestation and found that 27 subjects (10.3%) were positive for FVL compared to 11 controls (4.6%) [28]. The authors concluded that the FVL was significantly associated with RPL before 10 weeks gestation. The study of Grandone et al. also pointed out that the frequency of FVL is significantly higher among women with RPL and particularly associated with late events [30]. Furthermore, Rey et al., in a meta-analysis of the topic, found that FVL was associated with early (OR 2.01, 95% CI 1.13–3.58) and late (7.83, 2.83–21.67) RPL and late nonrecurrent fetal loss (3.26, 1.82–5.83) [10]. 

Our conclusions from the analysis of Palestinian women showed a statistically significant association between FVL and SB but not between FVL and RPL in the first or the second trimesters. These findings are in contrast with the findings of the European Prospective Cohort on Thrombophilia (EPCOT), which compared 571 women with thrombophilia with 395 control women. They defined miscarriage as a fetal loss in ≤28 weeks of gestation and stillbirth as a fetal loss in >28 weeks of gestation. The risk of fetal loss was higher in women with thrombophilia (OR 1.35). The OR was higher for SB than for miscarriage; 3.6 versus 1.27. The highest OR was for SB in women with multiple thrombophilias (14.3) compared with 2.0 for FVL alone, suggesting a synergistic effect [31]. 

In contrast to many studies which showed a significant association between FII and both early and late RPL [3, 10], our results did not show any association between FII and RPL (Table 2). In addition, the slight association with stillbirth (OR 1.48, [95% CI; 0.35–4.76]) was not statistically significant.

MTHFR is a metabolic disease which has been associated with arterial and venous TE [7]. It was of interest to study MTHFR in women who had RPL because placental infarcts have been associated with RPL [32]. However, MTHFR was underrepresented in the RPL group, which is in agreement with other reports [33, 34]. This may be attributed to folic acid supplementation, which is widely used especially in the first trimester of pregnancy. Folate status plays a crucial role in regulating homocysteine level in individuals homozygous for the MTHFR [33]. Therefore, it is reasonable to assume that folic acid supplementation may effectively silence the adverse effects of MTHFR, thus reducing the risk of RPL in women who are homozygous for this mutation.

It has been shown that women with concurrent polymorphism for FVL, FII, and MTHFR have an increased risk for TE and RPL than women with only one mutation [31]. We could not study this association because of the small number of these combinations in our data.

In conclusion, our study did not find a significant association between FVL, FII, and MTHFR and RPL in the first and second trimester. The only significant association was between FVL and SB.

## Figures and Tables

**Table 1:** Selected characteristics of cases and controls.

Characteristic	Cases *n* = 329	Controls *n* = 402	*P* value
Age (years)	28.1	28.9	.054
Parity (*n*)	1.16	3.15	.000
Consanguinity *n* (%)	109 (33.10%)	99 (24.60%)	.006
Family history of TE *n* (%)	62 (18.80%)	79 (19.70%)	.391
Diabetes Mellitus (*n*)	1 (0.3%)	3 (0.7%)	.210
Hypertension (*n*)	4 (1.2%)	2 (0.5%)	.142

**Table 2:** Number and prevalence of FVL, FII, and MTHFR in the cases and normal controls.

		FVL *n* (%)	FII *n* (%)	MTHFR *n* (%)
Cases	All cases *n* = 329	77(23.4%)	11 (3.3%)	21(6.4%)
	Consang. *n* = 109	28 (25.7%)	4 (3.7%)	8 (7.3%)
	Non-consang. *n* = 220	49 (22.3%)	7 (3.2%)	13 (5.9%)
	RPL *n* = 309	70 (22.7%)	11 (3.6%)	20(6.5%)
	SB *n* = 65	20 (30.8%)	4 (6.2%)	2(3.1%)

Controls	All controls *n* = 402	73 (18.2%)	17 (4.2%)	43(10.7%)
	Consang. *n* = 99	21 (21.2%)	7 (7.0%)	11 (11.1%)
	Non-consang. *n* = 303	52 (17.1%)	10 (3.3%)	32 (10.6%)

(i) *n* = number, (%) = prevalence, consang. = consanguineous, non-consang. = non-consanguineous.(ii) RPL: all cases excluding those with only stillbirth, SB: cases with stillbirth alone and stillbirth with first or second trimester loss.

**Table 3:** FVL, FII; and MTHFR association with RPL Subgroups, SB; and primary RLP.

Subgroups of RPL/SB	FVL	FII	MTHFR
	Odds ratio [95% CI]	Odds ratio [95% CI]	Odds ratio [95% CI]
Group A (*n* = 267)	**1.33**	**0.79**	**0.57**
	(0.892–1.989)	(0.305–0.909)	(0.297–1.044)
Group B (*n* = 50)	**1.13**	—	**0.53**
	(0.480–2.426)		(0.102–1.773)
Group C (*n* = 65)	2.0*	**1.49**	**0.27**
	(1.053–3.701)	(0.351–4.759)	(0.030–1.065)
Primary RPL (*n* = 168)	**0.719** (0.111–7.901)	**0.533** (0.071 + Inf)	**0.879** (0.119 + Inf)

Group A: women with repeated first-trimester miscarriage, Group B: women with repeated second-trimester miscarriage; Group C: women with SB, primary RPL: women with only first-trimester miscarriages and without a prior successful pregnancy.

*P = .028.

**Table 4:** Genotype and allele analysis of factor V Leiden G1691A, MTHFR C677T, and prothrombin (FII) G20210A.

Genotype	Allele				
FV G1691A	G/G (%)	G/A (%)	A/A (%)	G (%)	A (%)
Controls (*n* = 402)	329 (81.8)	69 (17.2)	4 (1)	727 (90.4)	77 (9.6)
Cases (*n* = 329)	250 (76)	72 (21.9)	7 (2.1)	572 (87)	86 (13)

MTHFR C677T	C/C (%)	C/T (%)	T/T (%)	C (%)	T (%)
Controls (*n* = 402)	182 (45.3)	177 (44)	43 (10.7)	541 (67.3)	263 (32.7)
Cases (*n* = 329)	145 (43.5)	151 (46.8)	33 (9.7)	441 (67)	217 (33)

FII G20210A	G/G (%)	G/A (%)	A/A (%)	G (%)	A (%)
Controls (*n* = 402)	385 (95.8)	17 (4.2)	0	787 (97.9)	17 (2.1)
Cases (*n* = 329)	318 (96.7)	11 (3.3)	0	647 (98.3)	11 (1.7)
